# Polypharmacy and the In Silico Prediction of Potential Body Proteins Targeted by These Drugs Among Hospitalized COVID-19 Patients With Cytokine Storm

**DOI:** 10.7759/cureus.48834

**Published:** 2023-11-15

**Authors:** Ghazwan A Raouf, Fouad K Mohammad, Muayad A Merza

**Affiliations:** 1 Department of Pharmacology, College of Pharmacy, University of Duhok, Duhok, IRQ; 2 Department of Physiology, Biochemistry and Pharmacology, College of Veterinary Medicine, University of Mosul, Mosul, IRQ; 3 College of Nursing, The American University of Kurdistan, Duhok, IRQ; 4 Department of Internal Medicine, University of Duhok, Duhok, IRQ

**Keywords:** target proteins, cytokine, extensive polypharmacy, multiple medications, coronavirus, sars-cov-2

## Abstract

Background and objective

Polypharmacy is prevalent in coronavirus disease 2019 (COVID-19) patients with severe disease. However, information on polypharmacy among COVID-19 patients who also suffer from cytokine storm is scarce. In light of this, the purpose of the present study was to assess the incidence of polypharmacy and in silico prediction of potential body proteins targeted by these drugs among hospitalized COVID-19 patients who were identified to have the additional burden of cytokine storm in the city of Duhok, Kurdistan Region, Iraq.

Methods

This was a cross-sectional observational study conducted from June 2021 to April 2022; the phenomena of major polypharmacy (six to nine medications) and excessive polypharmacy (≥10 medications) were documented among 33 (15 males and 18 females) COVID-19 patients with cytokine storm during their hospital stay (8-45 days) in Duhok, Kurdistan Region, Iraq. The SwissTargetPrediction program was utilized in silico to predict and identify human body proteins that could be potentially targeted by selected medications involved in polypharmacy.

Results

All patients had tested positive for COVID-19 via PCR testing, and they showed different signs and symptoms of the disease. None of the patients recovered and all of them deceased. All 33 patients received many therapeutic agents that ranged in number from eight to 20/patient during their hospital stay. The mean number of medications was 15 ± 3. We identified 2/33 (6%) patients with major polypharmacy (eight and nine) and 31/33 (94%) with excessive polypharmacy (15.5 ± 2.7). The total number of medications identified in polypharmacy was 37, excluding vitamins, minerals, and intravenous solutions. The frequency of medications administered was as follows: antibiotics (67, 13.7%), mucolytic agents (56, 11.5%), corticosteroids (54, 11%), anticoagulants (48, 9.8%), antiviral agents (41, 8.4%), antihypertensive agents (32, 6.5%), analgesics (28, 5.7%), antifungal drugs (27, 5.5%), antidiabetics (26, 5.3%), and other medications (2-19, 0.41-3.9%). Using the SwissTargetPrediction program, various drugs including antiviral agents involved in polypharmacy were found to target, in silico, body proteins at a prediction percentage that ranged from 6.7% to 40%.

Conclusions

Major and extensive polypharmacy conditions were identified in hospitalized COVID-19 patients suffering from cytokine storm. The severity of COVID-19 with cytokine storm, comorbidities, and hospitalization were key factors associated with polypharmacy in the patients. The SwissTargetPrediction web server is useful for predicting in silico potential human body protein targets that could possibly be sources of additional information on the adverse/toxic effects of polypharmacy medications administered concurrently. Further research in current medication protocols prescribed for advanced COVID-19 illness with cytokine storm is warranted to gain deeper insights into the topic.

## Introduction

The coronavirus disease 2019 (COVID-19) pandemic, caused by severe acute respiratory syndrome coronavirus 2 (SARS‑CoV‑2), has led to a significant global health crisis [1-3]. While the disease primarily affects the respiratory system, it could also lead to dysfunction in several organ systems, including the heart, liver, kidneys, and immune system [1-3]. This contributed to significant morbidity and the loss of millions of lives worldwide [1-4]. In severe forms of the disease, COVID-19 patients can experience an overwhelming cytokine storm [5,6]. This condition of systemic hyperinflammation is characterized by an increase in the release of chemokines and cytokines, including interleukins such as interleukine-1 and interleukine-6, interferons, tumor necrosis factor, colony-stimulating factors, the chemokine family, and growth factors [5,6]. Subsequently, the cytokine storm contributes to systemic deterioration and toxicity, disrupting the tricarboxylic acid cycle in various organ systems of COVID-19 patients [6,7]. The deterioration of COVID-19 cases involved with cytokine storm increases the demand for multiple medications and subjects the patients to polypharmacy, which might worsen the situation even more and potentially induce drug-drug interactions [8-12].

Polypharmacy is clinically associated with severe illnesses in which the patient receives more than five medications [13,14]. Within this context, polypharmacy with its potential drug-drug interactions and/or drug side effects and toxicity vulnerability poses a serious threat and might even increase the mortality rate in COVID-19 patients, especially those in an advanced stage of the illness with the need to be hospitalized [15,16]. Several studies have reported that polypharmacy is globally prevalent in COVID-19 patients with increased morbidity and mortality rates [17-20]. This is especially true in the elderly and hospitalized COVID-19 patients needing long-term care and medications that eventually result in multiple drug therapy [18,19,21]. While polypharmacy has been documented in severe cases of COVID-19 [20-22], there is scarce information on its incidence among patients who suffer from the additional burden of cytokine storm, which requires even more medications [10,11]. The purpose of the present study was to assess the incidence of polypharmacy and in silico prediction of potential body proteins targeted by these drugs in hospitalized COVID-19 patients with concomitant cytokine storm in the city of Duhok, Kurdistan Region, Iraq.

## Materials and methods

Patient selection and inclusion criteria

This was a cross-sectional observational study conducted to examine the phenomenon of polypharmacy among COVID-19 patients with cytokine storm. A total of 165 COVID-19 patients of both genders were hospitalized at two hospitals in Duhok, Kurdistan Region, Iraq (Duhok COVID-19, and Lalav Infectious Diseases Hospitals) from June 2021 to April 2022. The patients were diagnosed with COVID-19 based on nasopharyngeal and/or oropharyngeal swab collections, and the SARS-CoV-2 cases were confirmed by RNA extraction and real-time switch translation (RT)-PCR as per the WHO guidelines [23]. Of the 165 patients, 33 (15 males and 18 females) had severe COVID-19 clinical manifestations and they were diagnosed as having cytokine storm based on criteria including the worsening of the respiratory status and the three-fold elevation of at least two of the following markers: C-reactive protein, ferritin, D-dimer, lactate dehydrogenase, and cardiac troponin [24]. The cytokine storm in COVID-19 patients was assessed and confirmed by the above-mentioned criteria and consensus among the infectious disease physicians at the hospitals mentioned above. The demographic characteristics of the 33 COVID-19 cytokine storm patients and their signs and symptoms during the hospital stay were recorded. COVID-19 patients without cytokine storm were excluded from the study.

Ethical approval

Ethical approval to conduct the present study was obtained from the Committee of Post Graduate Studies, College of Science, University of Duhok, Kurdistan Region, Iraq, and from the Research Ethics Committee, Duhok Directorate General of Health, Duhok, Kurdistan Region, Iraq (24102021-10-10, dated October 24, 2021). Written consent was obtained from patients recruited for the study. They were informed about the study's purpose, the nature of data collection, and the expected outcomes of the study. All the information about the patients and the collected data were kept confidential.

Polypharmacy

For the purpose of this study, polypharmacy was defined as hospitalized COVID-19 cytokine storm patients receiving at least five medications on admission and continuing as such during their hospital stay (8-45 days) [25,26]. Polypharmacy was further classified into major polypharmacy (six to nine medications) and excessive polypharmacy (≥10 medications) [14]. The administration of medications to each patient was recorded, and the drugs were categorized according to their main clinical use and/or the expected outcomes, as follows: antibiotics, corticosteroids, mucolytic agents, anticoagulants, antipyretics/analgesics, antiviral agents, antidiabetics, proton pump inhibitors, antihypertensive agents, diuretics, anticholinergics, antacids, antipsychotics, sleep medications, antitussives, hypolipidemics, and pulmonary antifibrotic agents.

In silico prediction of human body protein targets by the drugs

We used the SwissTargetPrediction web server (http://www.swisstargetprediction.ch/) to predict and identify potential protein targets, in the human body, of selected diverse medications involved in polypharmacy. This online tool forecasts human protein targets for medications and could potentially predict drug efficacy and/or adverse action or toxicity [27,28]. The SwissTargetPrediction online program accepts canonical Simplified Molecular-Input Line-Entry System (SMILE) chemical structure formulae of drugs available at https://pubchem.ncbi.nlm.nih.gov/. Subsequently, these formulae were entered into the program in order to explore possible human body protein targets [27,28]. However, not all drug molecules are suitable for protein target prediction by the program because of the large molecular size limitations of some drugs such as insulin and vancomycin. The primary SwissTargetPrediction focus in the present study was on the antiviral agents remdesivir and favipiravir, which target, for example, the protease [29]. For the purpose of comparison, we selected eight additional drugs, with different structural formulae, as identified in the polypharmacy of the COVID-19 cytokine storm patients, to be subjected to SwissTargetPrediction analysis according to the availability of information in the program’s web server. A comprehensive SwissTargetPrediction review of all the 37 drugs listed in the present study was beyond the scope of the study.

Statistical analysis

Descriptive statistics were used to characterize the data, and the statistical software PAST 4.13 was used for statistical analysis (https://www.nhm.uio.no/english/research/resources/past/).

## Results

Table 1 shows the demographic characteristics of the 33 COVID-19 patients included in the study. They were 15 (45.5%) males (42-91 years old) and 18 (54.5%) females (34-80 years old) with a mean duration of hospital stay of 21.1 ± 10 and 22.2 ± 10.7 days, respectively. They were mostly overweight to severely obese (23/33, 69.7%), and at the time of hospital admission, the patients had preexisting disease conditions, which were as follows: hypertension (21, 63.6%), type 2 diabetes (20, 60.6%), peptic ulcer (13, 39.4%) chronic cardiac disease (8, 24.2%), hematological diseases (8, 24.2%), and other conditions (two to six, 6.1%-18.2%) (Table 1). Ten (30.3%) patients were vaccinated with COVID-19 vaccines. All the patients had tested positive for COVID-19 via PCR testing, and they all showed different signs and symptoms of COVID-19 disease (Table 2). Unfortunately, none of the patients recovered and they all deceased.

**Table 1:** Demographic characteristics of 33 hospitalized COVID-19 patients with cytokine storm COVID-19: coronavirus disease 2019; CPAP: continuous positive airway pressure therapy; PCR: polymerase chain reaction; SD: standard deviation

Variables	Males	Females	Total
N (%)	15 (45.5%)	18 (54.5%)	33 (100%)
Age, years, min-max	42-91	34-80	34-91
Duration of hospital stay, days, mean ± SD	21.1 ± 10.0	22.2 ± 10.7	21.7 ± 10.2
Duration of hospital stay, days, min-max	12-45	8-42	8-45
BMI, kg/m^2^, n (%)			
Underweight	2 (6.1%)	1 (3%)	3 (9.1%)
Healthy weight	3 (9.1%)	4 (12.1%)	7 (21.2%)
Overweight	5 (15.2%)	4 (12.1%)	9 (27.3%)
Obese	4 (12.1%)	7 (21.2%)	11 (33.3%)
Severely obese	1 (3%)	2 (6.1%)	3 (9.1%)
CPAP requirement, n (%)	15 (45.5%)	18 (54.5%)	33 (100%)
Preexisting medical conditions, n (%)			
Type 2 diabetes	9 (27.3%)	11 (33.3%)	20 (60.6%)
Arterial hypertension	9 (27.3)	12 (36.4%)	21 (63.6%)
Chronic cardiac disease	3 (9.1%)	5 (15.2%)	8 (24.2%)
Chronic pulmonary disease	2 (6.1%)	4 (12.1%)	6 (18.2%)
Bronchial asthma	1 (3%)	2 (6.1%)	3 (9.1%)
Peptic ulcer	6 (18.2%)	7 (21.2%)	13 (39.4%)
Thyroid disorders	3 (9.1%)	2 (6.1%)	5 (15.2%)
Chronic liver disease	1 (3%)	4 (12.1%	5 (15.2%)
Hematological disorders	2 (6.1%)	6 (18.2%)	8 (24.2%)
Cancer	1 (3%)	1 (3%)	2 (6.1%)
Vaccination, n (% )	6 (18.2%)	4 (12.1%)	10 (30.3%)
No vaccination, n (%)	9 (27.3%)	14 (42.4%)	23 (69.7%)
Positive COVID-19 PCR test, n (%)	15 (45.5%)	18 (54.5%)	33 (100%)
Polypharmacy, n (%)			
Major polypharmacy (6-9 medications)	2 (6%)	-	-
Excessive polypharmacy (≥10 medications)	31 (94%)	-	-
Outcome: deceased, n (%)	15 (45.5%)	18 (54.5%)	33 (100%)

**Table 2:** Signs and symptoms recognized in 33 hospitalized COVID-19 patients with cytokine storm COVID-19: coronavirus disease 2019

Signs and symptoms	Males, n (%)	Females, n (%)	Total, n (%)
Fever	14 (42.4%)	17 (51.5%)	31 (93.9%)
Cough	9 (27.3%)	9 (27.3%)	18 (54.5%)
Chill	3 (9.1%)	5 (15.2%)	8 (24.2%)
Sweating	1 (3%)	4 (12.1%)	5 (15.2%)
Dry mouth	5 (15.2%)	5 (15.2%)	10 (30.3%)
Chest tightness	14 (42.4%)	18 (54.5%)	32 (97%)
Chest pain	14 (42.4%)	13 (39.4%)	27 (81.8%)
Shortness of breath	15 (45.5%)	18 (54.5%)	33 (100%)
Headache	5 (15.2%)	9 (27.3%)	14 (42.4%)
Nausea	8 (24.2%)	11 (33.3%)	19 (57.6%)
Vomiting	3 (9.1%)	4 (12.1%)	7 (21.2%)
Diarrhea	6 (18.2%)	4 (12.1%)	10 (30.3%)
Stomachache	13 (39.4%)	13 (39.4%)	26 (78.8%)
Loss of appetite	11 (33.3%)	16	27 (81.8%)
Abdominal pain	4 (12.1%)	5 (15.2%)	9 (27.3%)
Constipation	7 (21.2%)	5 (15.2%)	12 (36.4%)
Epigastric pain	0 (0%)	1 (3%)	1 (3%)
Myalgia	15 (45.5%)	16 (48.5%)	31 (93.9%)
Joints pain	6 (18.2%)	4 (12.1%)	10 (30.3%)
Fatigue	15 (45.5%)	18 (54.5%)	33 (100%)
Loss of taste (ageusia)	1 (3%)	0 (0%)	1 (3%)
Bitter taste	1 (3%)	1 (3%)	2 (6.1%)
Loss of smell (anosmia)	5 (15.2%)	5 (15.2%)	10 (30.3%)

All 33 patients received several therapeutic agents that ranged in number from eight to 20/patient during their hospital stay, which ranged from eight to 45 days (Table 3). The mean number of polypharmacy medications/patient was 15 ± 3 with a median of 14 (Table 4). We identified 2/33 (6%) patients with major polypharmacy (eight and nine) and 31/33 (94%) with excessive polypharmacy (15.5 ± 2.7) (Tables 1, 3, 4). The total number of medications included in the polypharmacy was 37, excluding vitamins, minerals, and intravenous solutions (Table 4). The frequency of medications administered to the patients is shown in Table 4, which was as follows: antibiotics (67, 13.7%), mucolytic agents (56, 11.5%), corticosteroids (54, 11%), anticoagulants (48, 9.8%), antiviral agents (41, 8.4%), antihypertensive agents (32, 6.5%), analgesics (28, 5.7%), antifungal drugs (27, 5.5%), and antidiabetics (26, 5.3%). The frequency of administration of other medications ranged from two to 19 (0.41-3.9%).

**Table 3:** Medications administered to 33 hospitalized COVID-19 patients with cytokine storm *Major polypharmacy (6-9 medications); excessive polypharmacy (≥10 medications) COVID-19: coronavirus disease 2019

Patient no.	Medications administered	No. of medications^*^
1	Remdesivir, meropenem, vancomycin, levofloxacin, acetaminophen, methylprednisolone, enoxaparin, acetylcysteine, fluconazole, bromhexine, amlodipine, valsartan	12
2	Remdesivir, meropenem, vancomycin, acetaminophen, methylprednisolone, enoxaparin, acetylcysteine, famotidine, bromhexine, budesonide, ipratropium bromide, fluconazole, furosemide, insulin	14
3	Remdesivir, meropenem, vancomycin, acetaminophen, heparin, methylprednisolone, acetylcysteine, budesonide, fluconazole, haloperidol, melatonin, amlodipine, valsartan, hydrochlorothiazide, insulin, atorvastatin, omeprazole, bromhexine, spironolactone	19
4	Remdesivir, meropenem, levofloxacin, acetaminophen, dexamethasone, enoxaparin, budesonide, amlodipine, valsartan, insulin, atorvastatin	11
5	Remdesivir, favipiravir, levofloxacin, acetaminophen, methylprednisolone, enoxaparin, acetylcysteine, ipratropium bromide, fluconazole, nystatin, amlodipine, insulin, atorvastatin, bromhexine, theophylline, montelukast, ivy leaf product	17
6	Remdesivir, meropenem, levofloxacin, acetaminophen methylprednisolone, enoxaparin, acetylcysteine, insulin	8
7	Remdesivir, favipiravir, meropenem, vancomycin, acetaminophen, dexamethasone, methylprednisolone, acetylcysteine, famotidine, melatonin, amlodipine, valsartan, insulin, atorvastatin, enoxaparin, bromhexine, albumin, esomeprazole	18
8	Remdesivir, meropenem, levofloxacin, acetaminophen, methylprednisolone, acetylcysteine, budesonide, fluconazole, amlodipine, insulin, atorvastatin, bromhexine, enoxaparin, omeprazole, albumin	15
9	Remdesivir, meropenem, vancomycin, acetaminophen, methylprednisolone, enoxaparin, acetylcysteine, fluconazole, nystatin, clopidogrel, amlodipine, insulin, atorvastatin, ivy leaf product, omeprazole, albumin, furosemide	17
10	Remdesivir, vancomycin, levofloxacin, acetaminophen, heparin, methylprednisolone, acetylcysteine, budesonide, fluconazole, clopidogrel, amlodipine, insulin, atorvastatin, montelukast, theophylline, bromhexine, omeprazole	17
11	Remdesivir, vancomycin, levofloxacin, acetaminophen, methylprednisolone, enoxaparin, acetylcysteine, budesonide, fluconazole, clopidogrel, insulin, bromhexine, omeprazole	13
12	Remdesivir, favipiravir, meropenem, vancomycin, acetaminophen, methylprednisolone, acetylcysteine, famotidine, fluconazole, bromhexine, enoxaparin, omeprazole, bisoprolol, pirfenidone	14
13	Remdesivir, favipiravir, meropenem, vancomycin, methylprednisolone, acetylcysteine, budesonide, ipratropium bromide, fluconazole, clopidogrel, haloperidol, furosemide, insulin, bromhexine, enoxaparin, acetaminophen, omeprazole, albumin, atorvastatin, spironolactone	20
14	Remdesivir, favipiravir, meropenem, levofloxacin, acetaminophen, methylprednisolone, enoxaparin, acetylcysteine, amlodipine, valsartan, hydrochlorothiazide, insulin, atorvastatin, bromhexine	14
15	Remdesivir, meropenem, levofloxacin, acetaminophen, methylprednisolone, enoxaparin, acetylcysteine, amlodipine, insulin, atorvastatin, bromhexine	11
16	Remdesivir, vancomycin, levofloxacin, acetaminophen, methylprednisolone, acetylcysteine, budesonide, ipratropium bromide, haloperidol, enoxaparin, amlodipine, insulin, atorvastatin, bromhexine	14
17	Remdesivir, meropenem, vancomycin, acetaminophen, enoxaparin, methylprednisolone, acetylcysteine, budesonide, fluconazole, haloperidol, melatonin, amlodipine, insulin, atorvastatin, omeprazole, bromhexine, spironolactone	17
18	Remdesivir, favipiravir, meropenem, vancomycin, levofloxacin, acetaminophen, rivaroxaban, methylprednisolone, acetylcysteine, famotidine, budesonide, fluconazole, furosemide, amlodipine, insulin, atorvastatin, enoxaparin, bromhexine, pirfenidone	19
19	Remdesivir, meropenem, levofloxacin, acetaminophen, methylprednisolone, enoxaparin, acetylcysteine, budesonide, ipratropium bromide, haloperidol, amlodipine, insulin, atorvastatin, bromhexine	14
20	Remdesivir, favipiravir, meropenem, vancomycin, dexamethasone, methylprednisolone, acetylcysteine, famotidine, melatonin, amlodipine, valsartan, insulin, atorvastatin, bromhexine, enoxaparin, acetaminophen, albumin, esomeprazole	18
21	Remdesivir, meropenem, levofloxacin, acetaminophen, methylprednisolone, acetylcysteine, budesonide, fluconazole, insulin, bromhexine, enoxaparin, omeprazole, albumin	13
22	Remdesivir, meropenem, vancomycin, levofloxacin, acetaminophen, heparin, methylprednisolone, acetylcysteine, amlodipine, montelukast, theophylline, fluconazole, bromhexine	13
23	Remdesivir, favipiravir, meropenem, vancomycin, acetaminophen, methylprednisolone, enoxaparin, acetylcysteine, famotidine, bromhexine, budesonide, montelukast, theophylline, ipratropium bromide, fluconazole, furosemide, amlodipine, insulin, atorvastatin	19
24	Remdesivir, meropenem, acetaminophen, methylprednisolone, fluconazole, bromhexine, enoxaparin, albumin, esomeprazole	9
25	Remdesivir, favipiravir, levofloxacin, acetaminophen, methylprednisolone, montelukast, theophylline, enoxaparin, budesonide, ipratropium bromide, esomeprazole	11
26	Remdesivir, favipiravir, meropenem, levofloxacin, acetaminophen, methylprednisolone, acetylcysteine, fluconazole, nystatin, clopidogrel, enoxaparin, amlodipine, valsartan, hydrochlorothiazide, insulin, atorvastatin, ivy leaf product, omeprazole, albumin, furosemide	20
27	Remdesivir, favipiravir, meropenem, vancomycin, levofloxacin, acetaminophen, enoxaparin, apixaban, methylprednisolone, acetylcysteine, famotidine, nystatin, omeprazole, albumin	14
28	Remdesivir, favipiravir, vancomycin, levofloxacin, acetaminophen, heparin, methylprednisolone, acetylcysteine, budesonide, fluconazole, clopidogrel, insulin, theophylline, bromhexine, omeprazole, montelukast	16
29	Remdesivir, meropenem, vancomycin, acetaminophen, methylprednisolone, enoxaparin, acetylcysteine, famotidine, budesonide, ipratropium bromide, fluconazole, clopidogrel, bromhexine, albumin	14
30	Remdesivir, favipiravir, vancomycin, levofloxacin, acetaminophen, methylprednisolone, enoxaparin, acetylcysteine, budesonide, fluconazole, clopidogrel, amlodipine, insulin, atorvastatin, montelukast, theophylline, bromhexine, omeprazole	18
31	Remdesivir, meropenem, vancomycin, acetaminophen, methylprednisolone, acetylcysteine, fluconazole, nystatin, clopidogrel, insulin, enoxaparin, ivy leaf product, omeprazole, albumin, furosemide	15
32	Remdesivir, meropenem, vancomycin, dexamethasone, methylprednisolone, acetylcysteine, famotidine, melatonin, insulin, bromhexine, albumin, enoxaparin, acetaminophen, esomeprazole	14
33	Remdesivir, favipiravir, meropenem, vancomycin, acetaminophen, heparin, methylprednisolone, acetylcysteine, famotidine, bromhexine, budesonide, ipratropium bromide, fluconazole, furosemide, amlodipine, valsartan, insulin, atorvastatin	18

**Table 4:** Frequency of medications administered to 33 hospitalized COVID-19 patients with cytokine storm Vitamins, minerals, and intravenous solutions were not included in the polypharmacy COVID-19: coronavirus disease 2019

Medication	Frequency/33 patients	Medication classes	Frequency/33 patients	% of total frequencies (489)
Meropenem	26	Antibiotics	67	13.7
Vancomycin	22			
Levofloxacin	19			
Acetylcysteine	30	Mucolytic agents	56	11.5
Bromhexine	26			
Methylprednisolone	32	Corticosteroids	54	11.0
Dexamethasone	4			
Budesonide	18			
Heparin	14	Anticoagulants (Antithrombotic, Antiplatelet agents)	48	9.8
Enoxaparin	23			
Rivaroxaban	1			
Apixaban	1			
Clopidogrel	9			
Remdesivir	27	Antiviral agents	41	8.4
Favipiravir	14			
Amlodipine	20	Antihypertensive agents	32	6.5
Valsartan	8			
Hydrochlorothiazide	3			
Bisoprolol	1			
Acetaminophen	28	Analgesics/Antipyretics	28	5.7
Fluconazole	22	Antifungal agents	27	5.5
Nystatin	5			
Insulin	26	Antidiabetics	26	5.3
Esomeprazole	5	Proton pump inhibitors	19	3.9
Omeprazole	14			
Atorvastatin	19	Statin	19	3.9
Albumin	12	Plasma protein	12	2.5
Furosemide	8	Diuretics	11	2.25
Spironolactone	3			
Ivy leaf product	4	Antitussives	11	2.25
Theophylline	7			
Famotidine	10	H2 Blocker	10	2.04
Ipratropium bromide	9	Anticholinergics	9	1.84
Montelukast	7	Leukotriene receptor blocker	7	1.43
Haloperidol	5	Antipsychotics	5	1.02
Melatonin	5	Sleep regulators	5	1.02
Pirfenidone	2	Antifibrotic agents	2	0.41
Total	489	-	489	100

**Figure 1:**
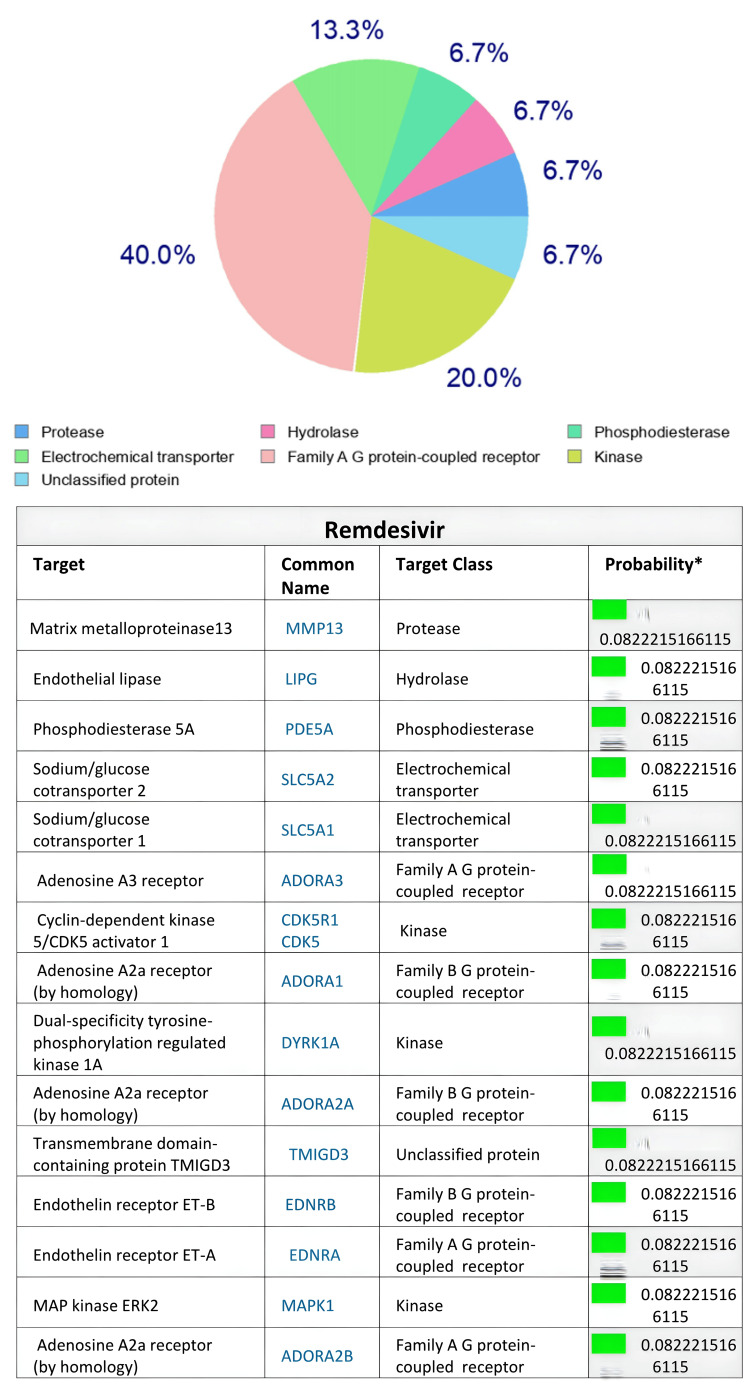
In silico SwissTargetPrediction web server prediction (%) of remdesivir’s human body protein targets

**Figure 2:**
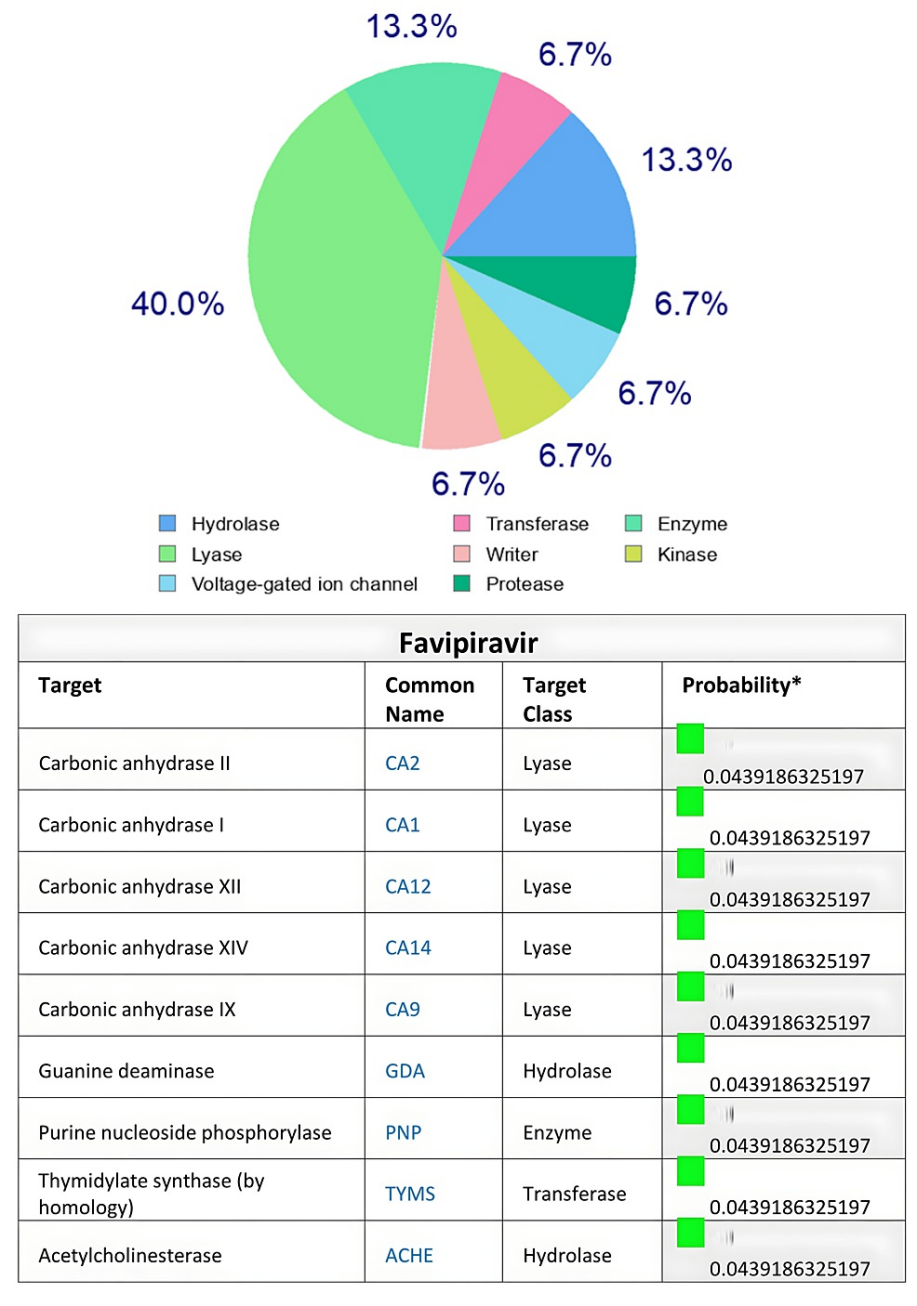
In silico SwissTargetPrediction web server prediction (%) of favipiravir’s human body protein targets

Based on the SwissTargetPrediction program, the human body proteins targeted by the two antiviral agents remdesivir and favipiravir after in silico evaluation are illustrated in Figures 1-2, respectively. Remdesivir targeted A-G receptor protein (40%), kinase (20%), and electrochemical transporter, followed by protease, hydrolase, phosphodiesterase, and unclassified protein (6.7% each) at p=0.0822 (Figure 1). On the other hand, favipiravir target classes included lyase (40%), hydrolase (13.3%), purine nucleoside phosphorylase (13.3%) and kinase, writer, proteases, and voltage-gated ion channels (6.7% each) at p=0.0439 (Figure 2). Examining eight other drugs included in the polypharmacy with the aid of SwissTargetPrediction for potential protein targets in the body indicated that these drugs target proteins to varying extents ranging from 6.7% to 40% (Tables 5, 6).

**Table 5:** In silico SwissTargetPrediction web server prediction of human body protein targets by various medications involved in polypharmacy recorded in COVID-19 patients with cytokine storm Detailed target outputs for remdesivir and favipiravir are presented in Figures 1-2. For a complete list of polypharmacy medications, see Tables 3-4 COVID-19: coronavirus disease 2019

Protein targets	% of proteins targeted by medications			
	Meropenem	Levofloxacin	Acetylcysteine	Prednisolone
Family AG protein-coupled receptor	33.3	26.7	13.3	6.7
Kinase	20	6.7	-	-
Protease	26.7	6.7	6.7	6.7
Lyase	13.3	-	-	-
Eraser	-	26.7	33.3	13.3
Cytochrome P450	-	-	-	6.7
Phosphodiesterase	-	13.3	-	-
Unclassified	-	-	-	-
Enzyme	-	13.3	26.7	-
Fatty acid-binding protein	-	-	-	6.7
Isomerase	-	-	-	-
Other cytosolic protein	-	-	-	-
Adhesion	-	-	-	-
Ligase	-	-	-	6.7
Oxidoreductase	-	6.7	-	-
Secreted protein	-	-	-	20
Electrochemical transferase	6.7	-	-	-
Hydrolase	-	-	-	-
Transferase	-	-	-	-
Writer	-	-	-	-
Voltage-gated ion	-	-	-	-
Phosphatase	-	-	20	6.7
Primary active transporter	-	-	-	-
Nuclear receptor	-	-	-	26.7

**Table 6:** In silico SwissTargetPrediction web server prediction of human body protein targets by various medications involved in polypharmacy recorded in COVID-19 patients with cytokine storm Continuation of Table 5 COVID-19: coronavirus disease 2019

Protein targets	% of proteins targeted by medications			
	Enoxaparin	Acetaminophen	Fluconazole	Omeprazole
Family AG protein-coupled receptor	-	-	-	6.7
Kinase	-	-	33.3	26.7
Protease	6.7	-	6.7	13.3
Lyase	13.3	40	13.3	-
Eraser	-	-	-	20
Cytochrome P450	-	6.7	13.3	-
Phosphodiesterase	-	-	-	6.7
Unclassified	6.7	6.7	6.7	-
Enzyme	-	33.3	20	13.3
Fatty acid-binding protein	-	-	-	-
Isomerase	6.7	-	6.7	-
Other cytosolic protein	26.7	-	-	-
Adhesion	26.7	-	-	-
Ligase	-	-	-	-
Oxidoreductase	26.7	-	-	-
Secreted protein	6.7	-	-	-
Electrochemical transferase	-	-	-	-
Hydrolase	-	-	-	-
Transferase	-	13.3	-	6.7
Writer	-	-	-	-
Voltage-gated ion	6.7	-	-	-
Phosphatase	-	-	-	-
Primary active transporter	-	-	-	6.7
Nuclear receptor	-	-	-	-

## Discussion

The COVID-19 patients included in the present study, who were suffering from cytokine storm with signs and symptoms of the disease, were hospitalized for varying durations (8-45 days). Being in such a critical condition, the patients were administered various therapeutic agents to combat the disease and its signs and symptoms, since a definitive cure for COVID-19 is not available [8-12]. However, not all the drugs were COVID-19-specific, as the patients were suffering from comorbidities, which can be characterized as a multimorbidity condition (Table 1), which demands additional medications. The COVID-19 patients in the present study eventually died during their hospital stay. This could be attributed in a collective manner to the severity of their condition [2,3,15,16], possible drug-drug interactions [18,19], complications exerted by the cytokine storm [5,10,11], as well as comorbidities and ineffectiveness of therapy [3,15-20]. These factors altogether quite possibly paved the way for polypharmacy in COVID-19 cytokine storm patients in the present study. However, we were not able to identify the benefit(s), if any, of multi-therapy in the COVID-19 patients with cytokine storm as all of them unfortunately deceased.

The types of polypharmacy identified in the present study were classified as major (6%) and excessive (94%). Similarly, polypharmacy findings have been reported by other investigators in COVID-19 patients with varying degrees of severity of the illness as well as comorbid conditions [15-20]. It certainly appears that hospitalization [16], old age [21], and the severity of COVID-19 illness [22] are the major factors that predispose patients to polypharmacy. The present study is unique in that we describe polypharmacy in hospitalized COVID-19 patients with cytokine storm, a condition that itself needs special care and additional supportive therapy [5,10,11], thereby increasing the possibility of extensive polypharmacy. Antibiotics were the most prevalent class of medications received by the COVID-19 patients in the present study. This finding aligns with another report showing elevated usage of antimicrobials in COVID-19 patients [19] because of the severity of the illness. It should be emphasized that polypharmacy is not solely linked to COVID-19 as many treatments of other acute or chronic illnesses potentially result in polypharmacy with adverse consequences [13,14].

Drug-drug interactions and subsequent adverse/toxic effects of the polypharmacy medications might occur in COVID-19 patients [8,12,15,16,18,19]. In the present study, we applied the SwissTargetPrediction online tool to detect protein targets in the human body that possibly interact with selected polypharmacy medications (including the antiviral agents) administered to COVID-19 patients. The SwissTargetPrediction findings, though not sufficiently comprehensive to include all the 37 medications reported in the present study, point to potential target body proteins shared by the polypharmacy drugs. The drugs administered to COVID-19 patients could possibly target these sites to produce adverse effects or even toxicity [28,29]. The SwissTargetPrediction analysis revealed that the antiviral agents, remdesivir and favipiravir used in COVID-19 patients in the present study, share common target proteins with other medications involved in the polypharmacy. These target proteins of remdesivir and favipiravir predicted by the SwissTargetPrediction included varying targeting percentages of A-G receptor protein, kinase, electrochemical transporter, protease, hydrolase, phosphodiesterase, lyase, hydrolase, purine nucleoside phosphorylase, writer, proteases, and voltage-gated ion channels classes. The target proteins predicted by SwissTargetPrediction for medications found in the polypharmacy group (Tables 5, 6) shared similar and additional target proteins, raising the possibility of adverse or toxic interactions (at the level of body proteins) of antiviral drugs with other medications co-administered to patients due to their critical conditions as a result of COVID-19 with cytokine storm. However, not all the medications shared similar targets, as predicted for acetaminophen (Table 6). Nevertheless, the SwissTargetPrediction tool could be an additional useful tool for drug-drug interaction used to predict possible adverse drug interaction/reactions in polypharmacy studies [13,14,29,30]. Further in silico and in vitro studies are warranted to explore the possibility of such interactions more deeply.

Limitations of the study

Adverse effects and toxicity of medications involved in polypharmacy that could have adversely affected the outcome were not determined in patients. We did not explore the possibility of drug-drug interaction using other drug-interaction programs. The benefits, if any, of multi-therapy in COVID-19 patients with cytokine storm could not be identified. Not all polypharmacy medications were subjected to STP, and a few were not accepted by the program, because of large molecular size or structural limitations.

## Conclusions

Major and extensive polypharmacy conditions were identified in hospitalized COVID-19 patients suffering from cytokine storm, and the beneficial effects of the therapeutic agents administered were unclear since all the patients died. The severity of COVID-19 with cytokine storm and comorbidities that needed hospitalization were key factors causing major and extensive polypharmacy conditions in these patients. The SwissTargetPrediction web server is a versatile tool for identifying and predicting in silico potential human body protein targets that could possibly be sources for information on additional adverse/toxic effects related to polypharmacy medications administered concurrently. We recommend further research on current medication protocols prescribed for advanced COVID-19 cases, especially those with the cytokine storm.
